# Interaction of various-sized particles in river flow

**DOI:** 10.1038/s41598-023-37460-y

**Published:** 2023-06-28

**Authors:** Niannian Fan, Qiang Zhong, Ruihua Nie, Xingnian Liu

**Affiliations:** 1 State Key Laboratory of Hydraulics and Mountain River Engineering, College of Water Resource & Hydropower, Sichuan University, Chengdu, 610065 China; 2 Department of Geography, University of British Columbia, Vancouver, V6T1Z2 Canada; 3 College of Water Resources and Civil Engineering, China Agricultural University, Beijing, 100083 China; 4 Beijing Engineering Research Center of Safety and Energy Saving Technology for Water Supply Network System, China Agricultural University, Beijing, 100083 China

**Keywords:** Hydrology, Fluid dynamics

## Abstract

Sediment transport is essential to the source-sink systems; however, the interaction between two complex multiscale nonlinear systems, turbulence of the river flow and wide size sediment, has heretofore restricted our understanding of sediment motion. We have conducted flume experiments deploying a video-based technique that records sediment transport rate of each particle size at 1 s resolution. The observations reveal detailed interactions between flow and particles of sizes ranging from 0.5 to 32 mm, such that small suspended particles (< ~ 5 mm) keep swirling in the wake vortices of the keystones (larger than 20 mm) until large to very-large-scale coherent structures destroy the wake vortices and bring the small particles downstream. Keystones destabilize consequently as the surrounding small and intermediate particles move, and in turn, a group of sheltered particles is entrained following the dislodging of the keystones. This heuristic model highlights the interactions of turbulence and different-sized particles.

## Introduction

In total, the rivers of the earth deliver more than 20 billion tons sediment to the oceans annually^1^. Sediment provides the major process linkage between hydrological factors^2^, channel bed roughness^3^, river channel morphology^4,5^ and sedimentation archives^6^. All modern aspects of habitat restoration, infrastructure planning, and pollution remediation require knowledge or prediction of the response of sediment to flooding^7^. Current sediment transport models attempt to predict transport rates from average descriptors of flow and bed material, such as the time-averaged shear stress and bulk grain size distribution, without explicit scale correlations^8–12^. Problematically, sediment transport, especially for river beds composing of poorly sorted particles, is a stochastic process^13^, and fluctuations of sediment transport rates orders larger than the mean have been shown in both field^14^ and experimental observations^15^. Highlighting that at timescales approximating the duration of a single flood these models fail to capture integral aspects of the transport process, in particular local interactions between flow and bed roughness elements.

**Figure 1:**
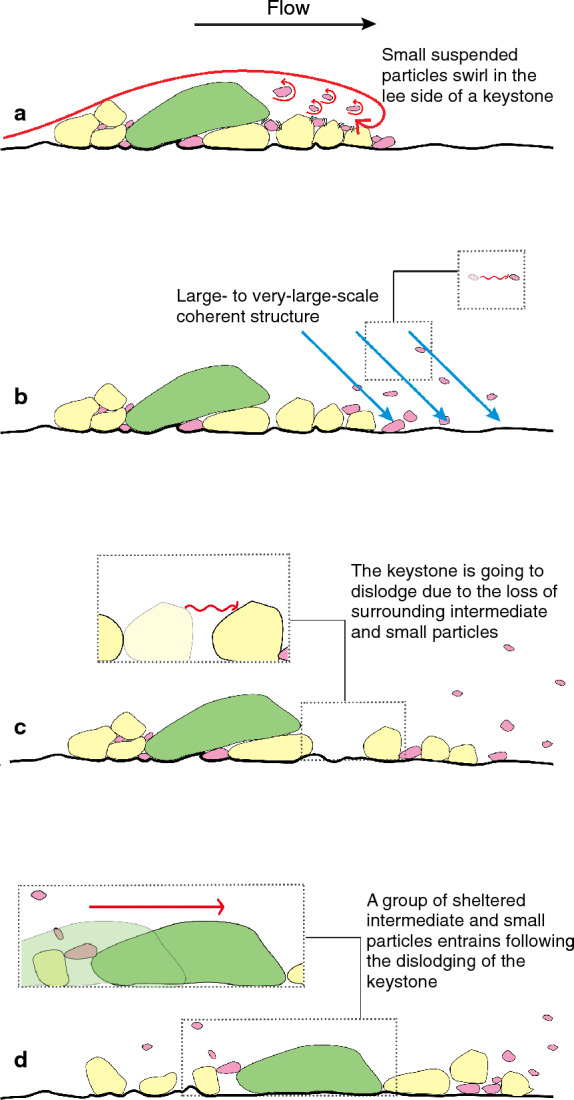
Sketch of flow and particle interactions in mixed size sediment transport. Flow is from left to right and the particles are grouped as small (pink), intermediate (yellow) and large (green) ones. (**a**) Small particles swirl in the lee of a large particle (key stone). (**b**) A group of small particles leaves the wake flow area in the lee of a key stone when a large to very-large-scale coherent flow structure passes by. (**c**) The intermediate particles are apt to be mobilized. (**d**) Intermediate and small particles supported by the keystone are mobilized after the keystone has become dislodged.

Consider a short section of nearly flat channel with a bed comprising poorly sorted natural sediment of mixed sizes exposed to a transporting flow (Fig. 1). At this scale, local interactions between near-bed turbulent structures and individual grains determine the entrainment behavior of particles^14,16–18^. This results in complex interactions between the flow and different-sized grains in transport, which may be described qualitatively using our conceptual model as Fig. 1. A large particle (> *D*_84_), which requires energetic flow to mobilize it, generates a downstream wake. The large particle shelters smaller particles, which are often trapped in small-scale turbulent vortices behind the large grain (Fig. 1a), seldom leave the wake flow area until a large coherent turbulent structure comes (Fig. 1b). As the intermediate particles become entrained (Fig. 1c), the large particle becomes unstable and is dislodged. As a result, particles immediately upstream of the large particle are mobilized (Fig. 1d). In this conceptual model, the cascade of interactions between stream flow and mixed-size particles determine the sediment transport processes, and temporal variability in sediment transport rates for each size group. As a result, we expect differently sized particles to show distinct time-dependent patterns of transport.

**Figure 2:**
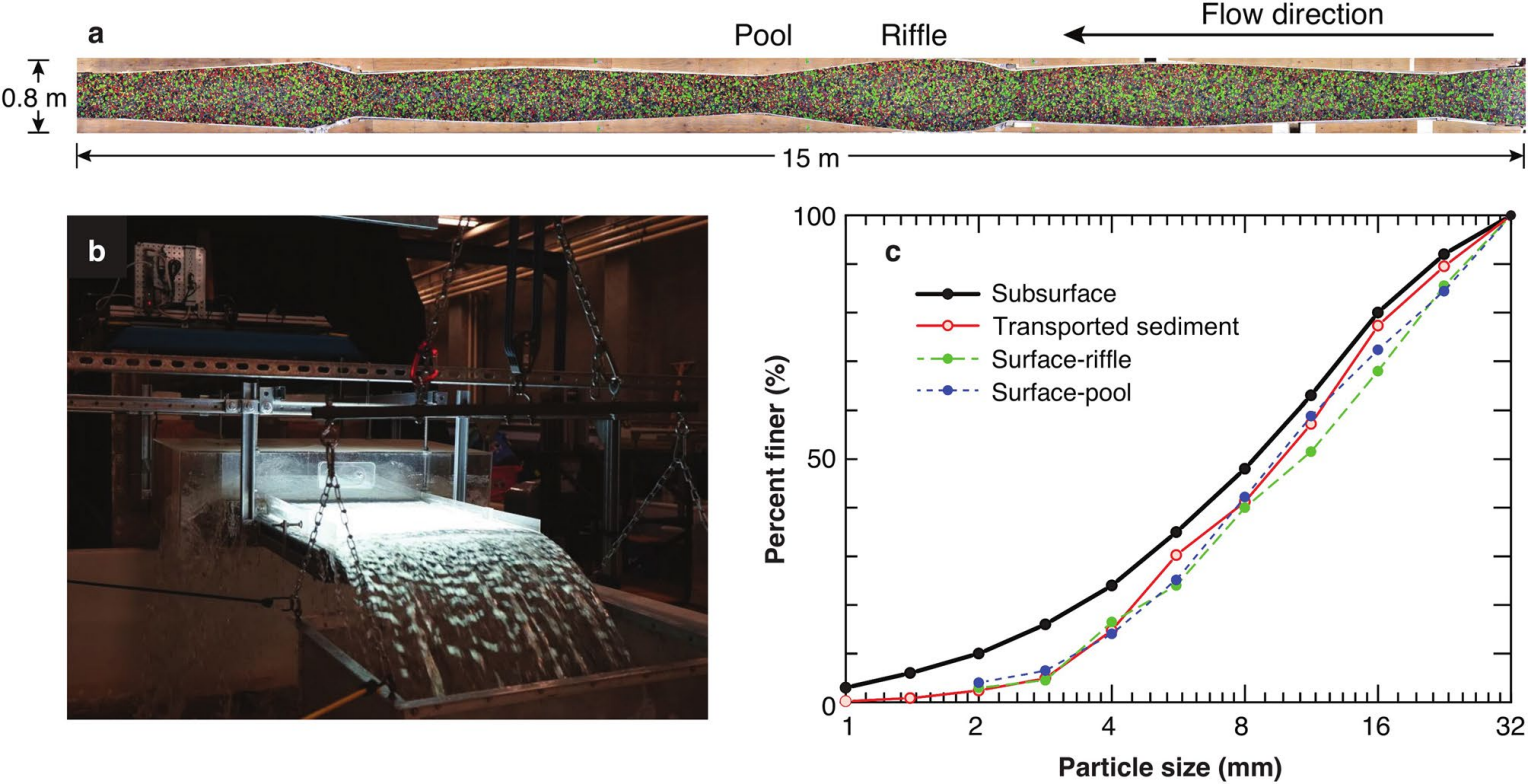
Overview of the experiment. (**a**) Image of the flume. The length of the flume was 18 m in total, but we had stitched photographs of only the middle 15 m. (**b**) Light table and basket at the outlet of the flume. (**c**) Size distribution for subsurface and feeding sediment (solid black line), transported sediment (solid red line), surface in riffle (dashed green line) and pool (dashed blue line) at the end of experiment.

**Figure 3:**
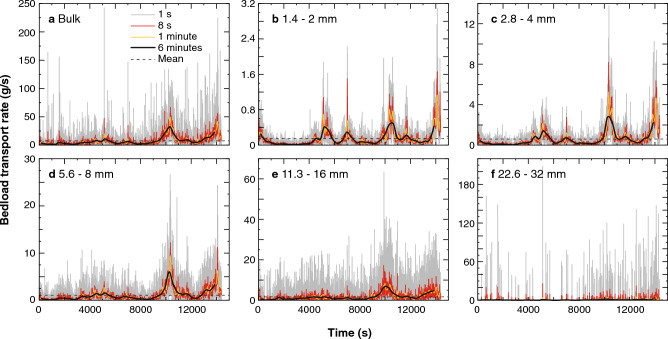
Time series of sediment transport rates collected for 4 h at sampling interval of 1 s, 8 s, 1 min and 6 min shown in grey, red, yellow and black solid lines respectively. The mean value is indicated as a black dashed line, note that *y*-axis scale varies for each plot. Subplot (**a**) is for the bulk sediment, and Subplots (**b**, **c**, **d**, **e** and **f**) are for particle size groups of 1.4–2 mm, 2.8–4 mm, 5.6–8 mm, 11.3–16 mm and 22.6–32 mm, respectively. Note that the longer the sampling interval, the smoother the sediment transport rates show.

To characterize the impact of the interactions between particles of various sizes in the proposed conceptual model, we conducted flume experiments with poorly sorted natural sediments (0.5–32 mm). The flume had a varying width (0.38–0.80 m) to force pool-riffle development^19^, in order to more closely recreate natural river conditions. Moreover, our experiment is unique in that a time series of sediment transport rates were measured for individual size classes from a video based light table at a 1 s temporal resolution (see Figs. 2b, 3 and Supplementary Information Fig. 2). Details of the experimental conditions are available in Methods. We hope this study sheds light on the direction of mixed-size sediment transport and provides further impetus for both theoretical and experimental studies. The particle individual or collective motion of various sizes were studied. In addition, the interactions of various-sized particles and turbulence flow structures were also discussed.

## Results

### Various sized sediment transport rates

Figure 3a displays the time series of bulk sediment transport rates at sampling intervals of 1 s, 8 s, 1 min and 6 min, respectively, along with the time-averaged mean. Figure 3b–f shows the sediment transport rates for each grain size subdivided into 0.5*φ* intervals (e.g., 1.4–2 mm, 2–2.8 mm, 2.8–4 mm, 4–5.6 mm…, 22.6–32 mm). The results show two distinct parts. First, as the sampling interval increases, the variability in sediment transport rate decreases. Second, larger particles show more intermittent transport characteristics, with the largest particles experiencing long periods of little motion followed by short lived periods of transport (see Fig. 3f).

As a stochastic time series, the variance of the sediment transport rates reflects the fluctuation magnitude, which decreases monotonically as the sampling interval increases. In our experiments, we expect that if each particle moves independently, a plot of variance and sampling interval in double-log space will have a slope of − 1^8,15,20^. Over a sampling interval in which particles movements are correlated (e.g. due to collective entrainment), a slope closer to 0 appears^15^. As a result, for a variance-sampling interval plot in log–log space, a change in slope reflects a changing pattern of motion across timescales. In addition, those plots differ for each particle size group, thus a cascade of interactions between each particle size group can be revealed. Moreover, if the timescales of a turbulence structure coincides with an observed time scale of particle motion it suggests this turbulence structure drives that pattern of particle motion.

**Figure 4:**
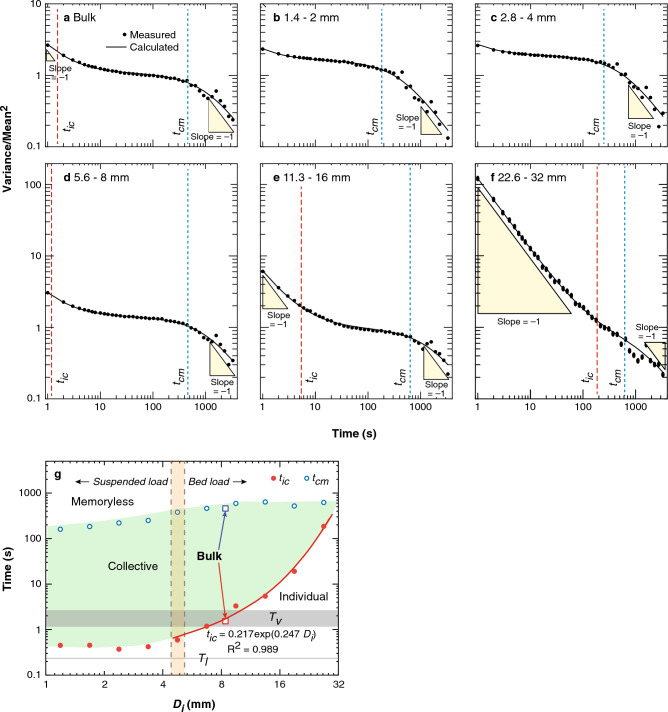
Normalized variance of sediment transport rates at different time scales. Subplot (**a**) is for the bulk sediment and Subplots (**b**–**f**) are for particle size groups of 1.4–2 mm, 2.8–4 mm, 5.6–8 mm, 11.3–16 mm and 22.6–32 mm, respectively. Two critical times, *t*_*ic*_ (red dashed line) and *t*_*cm*_ (blue dashed line) define the three regimes for variance decays at a slope of − 1, 0 and − 1 respectively. (**g**) Relations between critical times (*t*_*ic*_, red dots and *t*_*cm*_, blue dots) and particle sizes summarized from subplot (**a**–**f**), time scales of turbulence structures also show.

Figure 4a–f displays the relation between variance (scaled by the power of the mean value of sediment transport rate) and the sampling interval for the bulk and grain-size specific sediment transport rates. In Fig. 4a, the bulk transport rates can be divided into three distinct sampling interval ranges using the slope of the variance. At small sampling intervals, the variance decays approach a slope of − 1, suggesting that particle motions are individual and independent without correlation, which we define as individual time scale. In intermediate sampling intervals (2–400 s), however, the variance remains almost constant, particle motion is dominated by collective entrainment events, resulting in highly correlated sediment transport rates which we define as the collective timescale. At longer timescales, so many collective entrainment events occur that the memory between each event vanishes, we term this as the memoryless timescale. The time scale-variance relation could be fit using Eq. (1) (see “Methods” Section for details). Demarcating these timescales are two critical times, *t*_*ic*_ (separating individual and collective time scales) and *t*_*cm*_ (separating collective and memoryless time scales) (See Eq. 1 in “Methods” Section), marked as the red and blue dashed lines respectively.

### Interactions of turbulence and particles

Figure 4b–f allow for the comparison of these timescales across grain size classes, note that the 1 s sample time normalized variance for keystone (Fig. 4f) is at least one order larger than other particles (Fig. 4b–e). Figure 4g displays the relationship between particle size and the critical times (*t*_*ic*_ and *t*_*cm*_). It needs to note that, *D*_50_ plotted with both *t*_*ic*_ and *t*_*cm*_ for the bulk sediment fits the general trend of individual sediment size classes, which might support the convention that although the detailed transport processes for poorly sorted particles are more complex than for uniform particles^21–23^, *D*_50_ could serve as a proxy for the size of bulk sediment^24^.

## Discussion

As Fig. 4g, the relationship of *t*_*ic*_ and particle size shows two distinct segments with critical size of ~ 5 mm, corresponding to be the critical size boundary (4.4–5.4 mm) for suspended load and bed load (see “Methods” Section). *t*_*ic*_ keeps almost constant values (~ 0.4 s) for suspended load, which is in the range between *T*_*l*_ ~ 0.25 s and *T*_*v*_ ~ 2 s (Fig. 4g), supporting that large- to very-large-scale coherent flow structures (LSMs to VLSMs, in the order of 1 to 10 times the flow depth^25^) displace the suspended particles in the lee of keystones collectively^14^. Meanwhile, the relative weak wake vortices of the keystones, if drive downstream motion of suspended particles, only occasionally move them downstream individually, contributing individual motion scale smaller than* t*_*ic*_ (see “Methods” Section).

Figure 4g also shows the weak relationship between *t*_*cm*_ (separating collective and memoryless time scales) and particle size, which is interpreted to result from that following the dislodging of a keystone, a group of particles ranging over a wide size range sheltered by the keystone entrained simultaneously and collectively. However, keystones, the largest particles on the bed, are not sheltered by any larger particles, therefore *t*_*ic*_ and *t*_*cm*_ emerges, such that keystones do not experience collective entrainment.

The increasing of *t*_*ic*_ with the particle size for bed load illustrates the complex flow-particle and various-sized particle interactions. Firstly, the probability of occurrence of higher impulse events that can move larger particles decreases rapidly with the increasing of the particle size^21^. Secondly, particles interact with each other by granular contact network supported force chains^26,27^; with larger particles having more effective force chains^28^. The combination of these two factors may lead to the exponential growth of *t*_*ic*_.

It is important to address that the turbulence structure and mix-sized grain interactions we studied here is in a much smaller time scale than morphology adjustment or motion of bed forms^22^. For example, the periodic filling and eroding of pool-riffle morphology is in the scale of ~ 20 h (Supplementary Information Fig. 1), two or three orders longer than *t*_*cm*_ (order of 100 s). Nevertheless, the ways that particles interact at smaller spatial and temporal scales to drive riverbed morphology adjustments at larger scales warrants further study.

The results observed in this experiment are similar to the conceptual model elucidated in Fig. 1. Although our heuristic model is likely invalid during strong flow conditions capable of mobilizing all particle sizes, it is applicable for sediment transport in most gravel-bed alluvial rivers as shear stress during floods is usually only slightly larger than the critical shear stress for entrainment^29^. Exceptions to this generalization include outburst floods from dam breaks^30^ or very high sediment supplied conditions^31^, for those two conditions, we expect that particles move collectively and individual-grain transport does not exist.

## Conclusion

In this study, we observed poorly sorted sediment transport from flume experiments for steady transport condition. Size-specific transport rates at a temporal resolution of 1 s were obtained, allowing us to reveal grain/grain interactions and where possible, to reveal grain/turbulence flow interactions.

From the variation of the time series of size-specific sediment transport rates, we revealed both individual and collective motions at certain time scales. For smaller, suspendable sizes, the time scale boundary between individual and collective (*t*_*ic*_) corresponds to the time scale of large to very large turbulent fluctuations. For larger sizes in the mixture, *t*_*ic*_ increases with grain size. The upper limit of the collective transport time scale (*t*_*cm*_) is defined by the upper limit for individual transport of the coarsest grain size, suggesting that the time scale for the occasional entrainment of these keystone clasts defines the longest time variation of the bed, transport at longer time scales is uncorrelated and independent (memoryless) of shorter time scales. This time scale (roughly 500 s in our experiment) can be used to define the smallest time scale appropriate for modeling using transport formulas based on mean flow and bed properties.

We acknowledge that the time scales for turbulence flow structure were calculated from the flow condition, instead of direct measurement. Detailed in situ observations of turbulence and grain motion at various flow and sediment conditions (e.g., armoring and aggrading), or considering the sediment shape^32^ are required in future studies.

## Methods

### Experimental procedure

The flume^19,33^ is 18 m long with the bed inclined at a 1.5% slope. The width is variable, ranging from 0.38 to 0.80 m (Fig. 2a), allowing for morphodynamic development in different flume sections. The particle size distribution of the initial bed and the feeding sediment was the same, which was poorly sorted natural sand and gravel with sizes ranging from 0.5 to 32 mm, with a *D*_50_ of 8.4 mm. The sediment was sieved at 0.5*φ* intervals (e.g., 1.4–2 mm, 2–2.8 mm, 2.8–4 mm, 4–5.6 mm…, 22.6–32 mm), and different size classes were painted with different colors. Water was recirculated and discharge was controlled by a variable frequency pump, while sediment was fed to the flume inlet by a conveyor belt and collected with a basket trap at the flume outlet. See Table 1 for the brief information of the flume experiment.

**Table 1:** Brief information of the flume experiment.

Width (cm)	Depth (cm)	Slope	Flow rate (L/s)	Reynold number (10^4^)	Froude number	Sediment size (mm)	Sediment feed rate (g/s)
38–80	3.4–11	1.5%	50	6.26–13.2	1.56–3.19	0.5–32	10.9

Flow discharge was kept constant at 50 Liters/s. Riffles (in the wide sections) and pools (in the narrow sections) were formed from an initially levelled bed, after running the flume for 81 h without sediment feed until sediment load transport rate was very low (0.079 g/s). After these low transport rates were achieved (indicating a stable bed), sediment with a rate of 10.9 g/s (dashed line in Supplementary Information Fig. 1) was fed at the flume inlet and these conditions were run for 68.5 h (Supplementary Information Fig. 1). At approximately 36 h, the sediment transport rate became equivalent to the feeding rate indicating that quasi steady-state conditions had been reached. Detailed sediment transport rates were obtained from a video-based light table^34^ (see Supplementary Information Fig. 2, the interval of 42.5–46.5 h marked grey in Supplementary Information Fig. 1), at time scales of 1 s for different sizes at 0.5*φ* interval. Although the cutoff value of the smallest particle sizes was 0.5 mm, transport rate data from the light table were not reliable for particles < 1 mm^20^ so they were not used for further analysis.

From 42.5 to 46.5 h, the total sediment mass from the video-based method was calculated as 109.4 kg, while 110.7 kg of sediment was collected from the basket at the outlet of the flume, indicating a very small (1.2%) under-estimation bias. Also, the particle size distributions derived from the video-based method and sieving from basket collected sediment are very close (Supplementary Information Fig. 3), indicating that the video-based method performed well.

### Model for fitting the three ranges of variance–time relations

Rearranging from the model shown in reference^15^, Eq. (1), the function between time scale and (normalized) variance of sediment transport rate, was obtained as:

1 $${\text{Var/Mean}}^{2} \left( {\Delta t} \right) = \frac{a}{\Delta t}\left\{ {\frac{{2t_{{{\text{cm}}}} }}{{t_{{{\text{ic}}}} }}\left[ {1 - \frac{{t_{{{\text{cm}}}} }}{\Delta t}\left( {1 - e^{{ - \frac{\Delta t}{{t_{{{\text{cm}}}} }}}} } \right)} \right] + 1} \right\}$$

where the variance (Var) was normalized by the square of the mean (Mean) value, *t*_*ic*_ and *t*_*cm*_ are the critical times dividing individual, collective and memoryless time scales, respectively. *a* is a constant coefficient for calibration.

We acknowledge that for particles smaller than 5.6 mm, the *t*_*ic*_ are smaller than the resolution of sediment transport rate time series in our experiment (1 s) as shown in Fig. 4b–c, and thus the values of *t*_*ic*_ are obtained by extension of the model as Eq. (1). However, the trends for − 1 slope when time scales approach to 1 s are obvious as Fig. 4b and c, as a result, we consider the calibrated values of *t*_*ic*_ from Eq. (1) are reliable.

### Estimation of flow structure time scales

The period of the very-large-scale coherent structures, *Tv*, is calculated as reference^35^

2 $$T_{v} = 5.7B^{0.6} h^{0.4} {\text{/u}}$$

where *B*, *h* and *u* are the channel width, depth and average cross-section velocity, respectively. In pool and riffle sections, the values of *B* are 0.38 and 0.80 m and the value of *h* are 13.5 and 8.4 cm, the values of *u* are calculated to be 0.97 and 0.76 m/s, respectively from the constant discharge Q = 50 L/s. As a result, *T*_*v*_ is calculated to be in the range of 1.5–2.4 s.

The period of the large-scale coherent structure is calculated as reference^17^

3 $$T_{l} = 2{\text{h/u}}$$

and *T*_*l*_ is calculated to be in the range of 0.22–0.27 s.

The period of wake vortices shedding of the keystones is calculated as^36,37^

4 $$T_{w} = D_{k} /\left( {S_{t} u} \right)$$

where *D*_*k*_ is the dimension of keystones (~ 25 mm), *S*_*t*_ is the Strouhal number, and here *S*_*t*_ = 0.19 as the keystone Reynolds number Re_*d*_ is in the range of 1.8–2.5 × 10^4^. As a result, *T*_*w*_ is calculated to be in the range of 0.11–0.14 s.

### Estimation of size boundary for bed load and suspended load

The threshold for sediment suspension was calculated based on the ratio of shear velocity u_*_ and particle terminal settling velocity *w*_*s*_ as u_*_/*w*_*s*_ = 0.4, particles were suspended load or bed load if u_*_/*w*_*s*_ > 0.4 or < 0.4, respectively^38^, given u_*_ = 11.5–12.9 cm/s in our experiment, we calculated the particles in the threshold for suspension had *w*_*s*_ in the range of 28.8–32.2 cm/s.*w*_*s*_ was the function of particle size *D* as reference^39^

5 $$w_{s} = \frac{{RgD^{2} }}{{C_{1} \nu + \sqrt {0.75C_{2} RgD^{3} } }}$$

where *R* is submerged specific gravity (1.65 for the particles in our experiment), *g* is gravitational acceleration, which equals to 9.81 m/s^2^, *C*_1_ = 20 and *C*_2_ = 1.1 are constants for natural particles, and *ν* is the kinematic viscosity of water, which equals to 10^–6^ m^2^/s.

Then critical size for particle suspension in our experiment was calculated to be in the range of 4.4–5.4 mm.

## Supplementary Information

Supplementary Information.

## Data Availability

All data are available in the main text and the Supplementary Material.
