# Supplementary material for: Interaction of various-sized particles in river flow
Source: Sci Rep. 2023 Jun 28;13:10503. doi: 10.1038/s41598-023-37460-y (PMC10307775; doi:10.1038/s41598-023-37460-y)
Supplement: Supplementary file 1 — Supplementary Information. [file 41598_2023_37460_MOESM1_ESM.docx]

Supplementary

# Interaction of various-sized particles in river flow

Niannian Fan^1, 2^*, Qiang Zhong^3, 4^, Ruihua Nie^1^ and Xingnian Liu^1^

1. State Key Laboratory of Hydraulics and Mountain River Engineering, College of Water Resource & Hydropower, Sichuan University, Chengdu 610065, China
2. Department of Geography, University of British Columbia, Vancouver V6T1Z2, Canada
3. College of Water Resources and Civil Engineering, China Agricultural University, Beijing 100083, China.
4. Beijing Engineering Research Center of Safety and Energy Saving Technology for Water Supply Network System in China Agricultural University, Beijing 100083, China


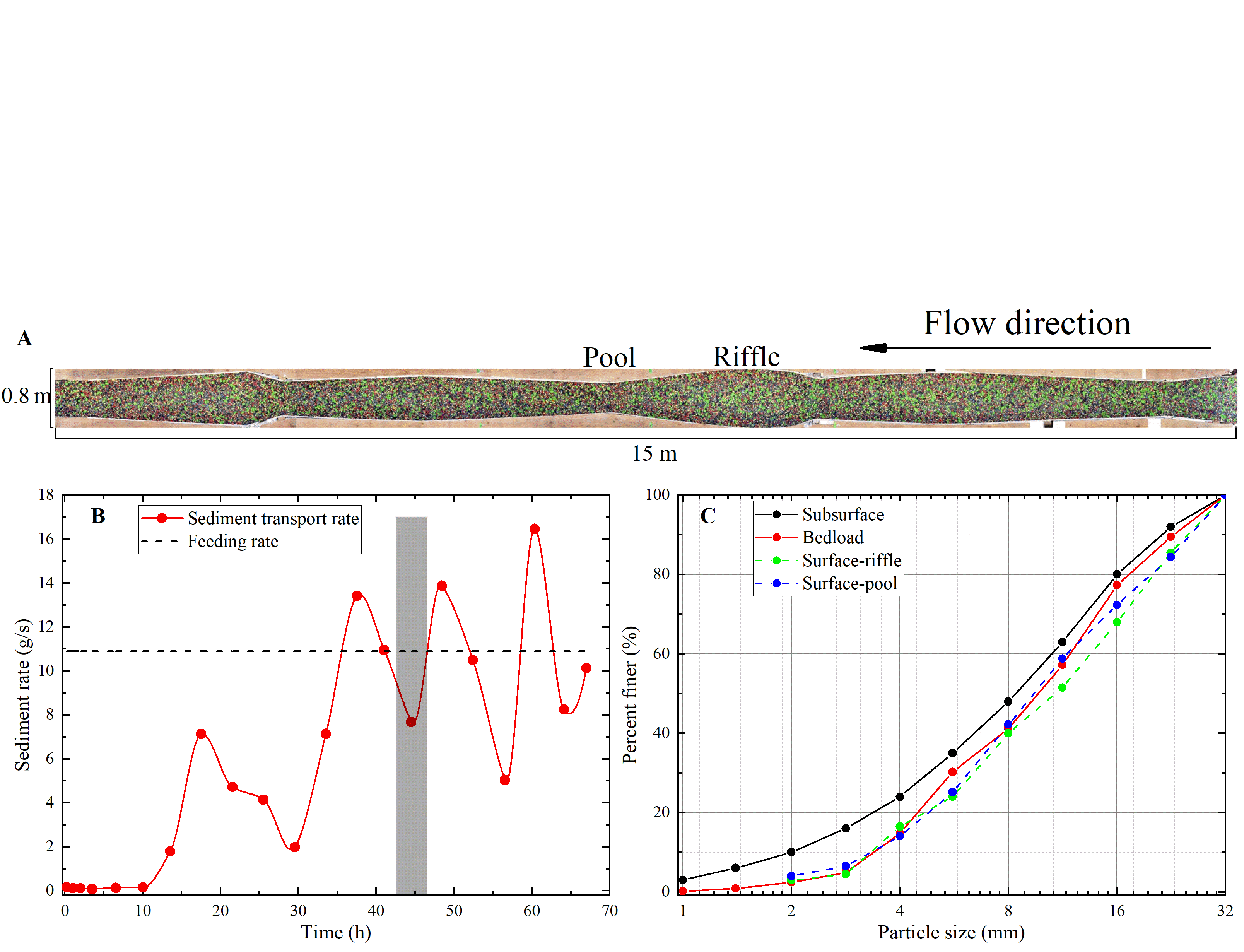


**Supplementary Information Figure 1.** Feeding rate (black dashed line) and the sediment transport rate (red solid line) collected from the end of the flume, note that at about 36 h, the sediment transport rate met the feeding rate and it was determined that quasi-steady conditions had been reached. The 1 s resolution video-based bed load transport measurement for difference size group was analyzed during the 42.5-46.5 h marked as a grey band.


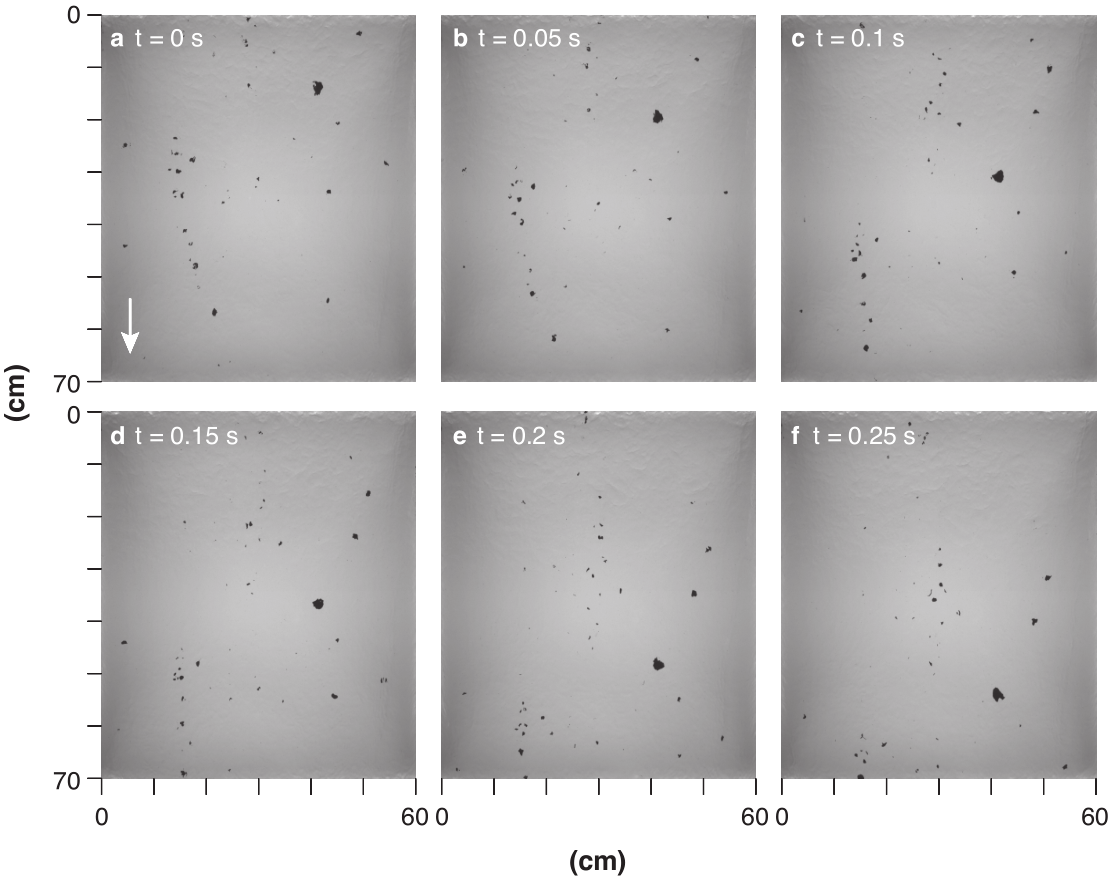


**Supplementary Information Figure 2.** Frames distracted from light table video. The frame is 70 cm long, 60 cm wide, and the flow is from top to bottom, time interval for each frame is 0.05 s.

**Supplementary Information Figure 3.** Comparison of particle size distributions between sieving from the basket collected sediment as black line and light table as red table, indicating the two series are very similar.
